# Selective Enrichment of Omega-3 Fatty Acids in Oils by Phospholipase A1

**DOI:** 10.1371/journal.pone.0151370

**Published:** 2016-03-15

**Authors:** Tushar Ranjan Moharana, Avinesh R. Byreddy, Munish Puri, Colin Barrow, Nalam Madhusudhana Rao

**Affiliations:** 1 CSIR-Centre for Cellular and Molecular Biology, Uppal Road, Hyderabad, India; 2 Centre for Chemistry and Biotechnology, Deakin University, 75 Pigdons Road, Waurn Ponds, Victoria 3220, Australia; National Research Council of Italy, ITALY

## Abstract

Omega fatty acids are recognized as key nutrients for healthier ageing. Lipases are used to release ω-3 fatty acids from oils for preparing enriched ω-3 fatty acid supplements. However, use of lipases in enrichment of ω-3 fatty acids is limited due to their insufficient specificity for ω-3 fatty acids. In this study use of phospholipase A1 (PLA1), which possesses both *sn-1* specific activity on phospholipids and lipase activity, was explored for hydrolysis of ω-3 fatty acids from anchovy oil. Substrate specificity of PLA1 from *Thermomyces lenuginosus* was initially tested with synthetic *p*-nitrophenyl esters along with a lipase from *Bacillus subtilis* (BSL), as a lipase control. Gas chromatographic characterization of the hydrolysate obtained upon treatment of anchovy oil with these enzymes indicated a selective retention of ω-3 fatty acids in the triglyceride fraction by PLA1 and not by BSL. ^13^C NMR spectroscopy based position analysis of fatty acids in enzyme treated and untreated samples indicated that PLA1 preferably retained ω-3 fatty acids in oil, while saturated fatty acids were hydrolysed irrespective of their position. Hydrolysis of structured triglyceride,1,3-dioleoyl-2-palmitoylglycerol, suggested that both the enzymes hydrolyse the fatty acids at both the positions. The observed discrimination against ω-3 fatty acids by PLA1 appears to be due to its fatty acid selectivity rather than positional specificity. These studies suggest that PLA1 could be used as a potential enzyme for selective concentrationof ω-3 fatty acids.

## Introduction

Based on several prospective and retrospective studies it has been shown that consumption of fish oils has health benefits, mainly in cardiovascular events, [1,2]. Most of the benefits of fish oil were attributed to the polyunsaturated omega-3 (ω-3) fatty acids, namely, eicosapentaenoic acid (EPA) and docosahexaenoic acid (DHA) [3]. The American Heart Association recommends consumption of 1g/day of ω-3 fatty acids by patients with coronary heart diseases [4]. ω-3 fatty acids constitute approximately 30% of the total fatty acids in natural fish oils. Concentrated esters of ω-3 fatty acids have been formulated to deliver higher amounts of EPA and DHA per dose to patients [1]. However, conversion of fatty acids to ethyl esters followed by fractional distillation and urea concentration damages these oxidatively sensitive ω-3 fatty acids. Also, re-esterification to triacylglycerides requires further processing of these fatty acids, which results in statistical distribution of fatty acids on the glycerol backbone [5].

To overcome these challenges, lipases were employed to selectively hydrolyse ω-3 fatty acids [6].Triglycerides from natural fish oils are complex in composition [7]. Besides containing several kinds of fatty acids, mainly saturated, monounsaturated and polyunsaturated, the fatty acids are also non-uniformly distributed on the glycerol backbone. ^13^C NMR spectral studies on anchovy fish oil shows that DHA in more abundant at sn-2 than at sn-1 and -3 positions, while distribution of EPA is more abundant at sn-1 and sn-3 position compared to sn-2 position [8]. The non-uniform distribution of fatty acids on glycerol further confounds their selective hydrolysis by lipases.

Lipases have been tested for selective concentration of ω-3 fatty acids from fish oils either by fish oil hydrolysis or by selective esterification [8–11]. Lipases possess some important properties such as partial selectivity towards chain length and position of fatty acid in glycerol and they also discriminate between fatty acids with single and multiple double bonds. These properties of lipases make them as suitable candidates for enzymatic concentration of ω-3 fatty acids [12]. Most lipases preferentially hydrolyse saturated and mono-unsaturated fatty acids from triglycerides and discriminate against ω-3 fatty acids, apparently due to the presence of double bonds that cause steric hindrance in the active site of a lipase [13,14]. Most lipases preferentially hydrolyse EPA over DHA, probably due to the presence of an additional double bond located closer to the ester bond in DHA [8,15]. In a study five lipases were tested for specificity in the hydrolysis of fish oil and fatty acid esters as controls. Discrimination against EPA and DHA was observed with fatty acid esters but not with fish oils [6]. Another study on lipase mediated fish oil hydrolysis suggested that hydrolysis is biased towards the chemical nature of the fatty acid rather than to their abundance at a given position on glycerol [8]. In another study, pancreatic lipase was observed to preferentially hydrolysed docosapentaenoic acid(DPA) over EPA and DHA [16].

Phospholipase A1specifically hydrolyse phospholipids to release fatty acids at the sn-1 position and releases a 2-acyl lysophospholipid. Functions of PLA1 are not clearly established and some PLA1s were reported to show lipase-like activity [17,18]. Lipases that show phospholipase activity have been extensively investigated but not the reverse [19].The catalytic mechanism between lipases and phospholipases is identical; however, the specificity emerges from the active site properties. Studies have indicated that lipases with a short lid and a short β9 loop are more suitable to accommodate polar phospholipids [20]. To investigate the ability of a phospholipase in regioselective hydrolysis of triglycerides, we chose PLA1 in this study since it is more likely to be selective for the sn-1,3 position in triglycerides.

Phospholipase A1 used in this study is a recombinant enzyme manufactured by Novozymes. This enzyme was observed to hydrolyse both phospholipids and triglycerides with a single active site [17,21]. The phospholipase activity of this enzyme is utilized in commercial degumming of vegetable oils and for modifying phospholipids [22,23]. The lipase activity of PLA1 was used in organic synthesis of structured lipids [17]. PLA1 also successfully immobilised on different nanoparticles to further improve its catalytic properties [17,24,25]. In this study, we used PLA1 for concentration of ω-3 fatty acids from anchovy fish oil. The ability of PLA1 to concentrate ω-3 fatty acids into the triglyceride portion was investigated by examining the preferential fatty acid hydrolysis using gas chromatography and ^13^C NMR spectroscopy.

## Materials and Methods

### Chemicals

Bleached anchovy oil was supplied by Ocean Nutrition Canada (Canada). The major fatty acid composition of anchovy oil was determined by gas chromatography analysis [8]. Phospholipase A1 (Lecitase Ultra^®^), Dioleoyl-2-palmitoylglycerol,gum Arabic and methyl nonadecanoate and gas chromatography standards were procured from Sigma-Aldrich(Castle Hill, Australia). TLC plates were procured from Merck, India. All the buffers were made using analytical grade chemicals. All solvents used were either analytical grade or higher.

### Methods

#### Hydrolysis of anchovy oil

Reaction mixture containing 50 mMTris, 25 mM CaCl_2_, 5% (w/v) gum arabic at pH 8.00 and 5% (v/v) anchovy oil were emulsified by sonication. 100 units of PLA1 were added and the reaction was carried at 37°C with constant stirring in the presence of nitrogen gas. Rate of reaction was monitored by pH stat (Ω Metrohm 718 STAT Tritino) by titrating with 1 M NaOH. 2 ml of sample was drawn at different time points and free fatty acid was separated from glyceride by solvent extraction method as described earlier [26]with modifications. Enzyme hydrolysate (2 ml) was dissolved in 5 ml ethanolic (30%) KOH (0.5M) to solubilise free fatty acids as potassium salts. Insoluble glycerides in the alkaline ethanolic fraction were extracted with 5 ml of hexane. The remaining ethanolic fraction containing free fatty acids was extracted with 5 ml of hexane after adding 0.5 ml 12N HCl. The efficiency of extraction was confirmed by TLC. Solvents in the extracts were removed by evaporation in the presence of nitrogen gas and the glyceride fractions obtained were stored in an airtight polypropylene tube at -20°C till further analysis. All procedures were done under nitrogen environment to reduce the exposure of the sample to air.

#### IATROSCAN analysis

Both the unhydrolysed and hydrolysed portions of the fish oil were analysed by capillary chromatography with flame ionization detector (Iatroscan MK5, Iatron Laboratories Inc., Tokyo, Japan). The Iatroscan settings were: air flow rate, 200 ml/min; hydrogen flow rate, 160 ml/min and scan speed, 30s/scan. Under these conditions, the chromarods were cleaned by scanning twice before applying samples. One microliter of each lipid fraction in hexane was spotted onto the rods with the aid of an auto pipette along the line of origin on the rod holder and rods were developed for 22 minutes in a solvent tank containing hexane/diethyl ether/acetic acid (60:17:0.2, vol/vol/vol). TLC standards purchased from Nu-Chek Prep were used to identify each lipid class [8].

#### Methylation and GC analysis

5 μL (both free fatty acid and glyceride) sample was methylated by using acetyl chloride in methanol as described by Christie et al [27] with minor modifications. 1 ml toluene was added to the methylation tubes followed by the addition of 200 μl (5mM)of internal standard, methyl nonadecanoate (C19:0) and 200 μl (1mM) of butylatedhydroxytoluene (BHT). 2ml of acidic methanol (prepared by adding 10% acetyl chloride in methanol drop wise on ice bath) was added to the tube and kept for overnight incubation at 50°C. Fatty acid methyl esters (FAMEs) were extracted into hexane. The hexane layer was removed and dried over anhydrous sodium sulphate. FAMEs were concentrated by drying using nitrogen gas and analysed by gas chromatography. Hydrolysed and unhydrolysed fish oil was analysed using gas chromatography (Agilent 6890) with flame ionisation detector (FID), equipped with a Supelcowax 10 capillary column (30 m, 0.25 mm i.d., 0.25 μm film thickness; Supelco). Helium was used as the carrier gas at a flow rate of 1.5 ml min^-1^. The injector was maintained at 250°C and a sample volume of 1 μl was injected. Fatty acid peaks were identified by comparing with data on retention times of external standards (Sigma-Aldrich, St. Louis, MO, USA) and corrected using theoretical FID response factors [28]. Peaks were quantified with Chemstation chromatography software (Agilent Technologies, Santa Clara, CA, USA).

#### Analysis of positional distribution of fatty acid by NMR

300 μl of fish oil was dissolved in 1 ml of CDCl_3_ (99.8% pure). NMR spectra were collected by using Bruker 600MHz NMR machine. Peak corresponding to different fatty acids at different positions were assigned as described earlier [29]. Relative quantification was done by comparing the area under each peak. Quantitative ^13^C NMR spectra of the unhydrolysed oils were recorded under continuous ^1^H decoupling at 24°C.In order to quantify the residue of each fatty acid at different positions, peak area ratios were analysed by integration and presented in percentages [8].

## Results

### Esterase activity of Phospholipase A1

**Fig 1:**
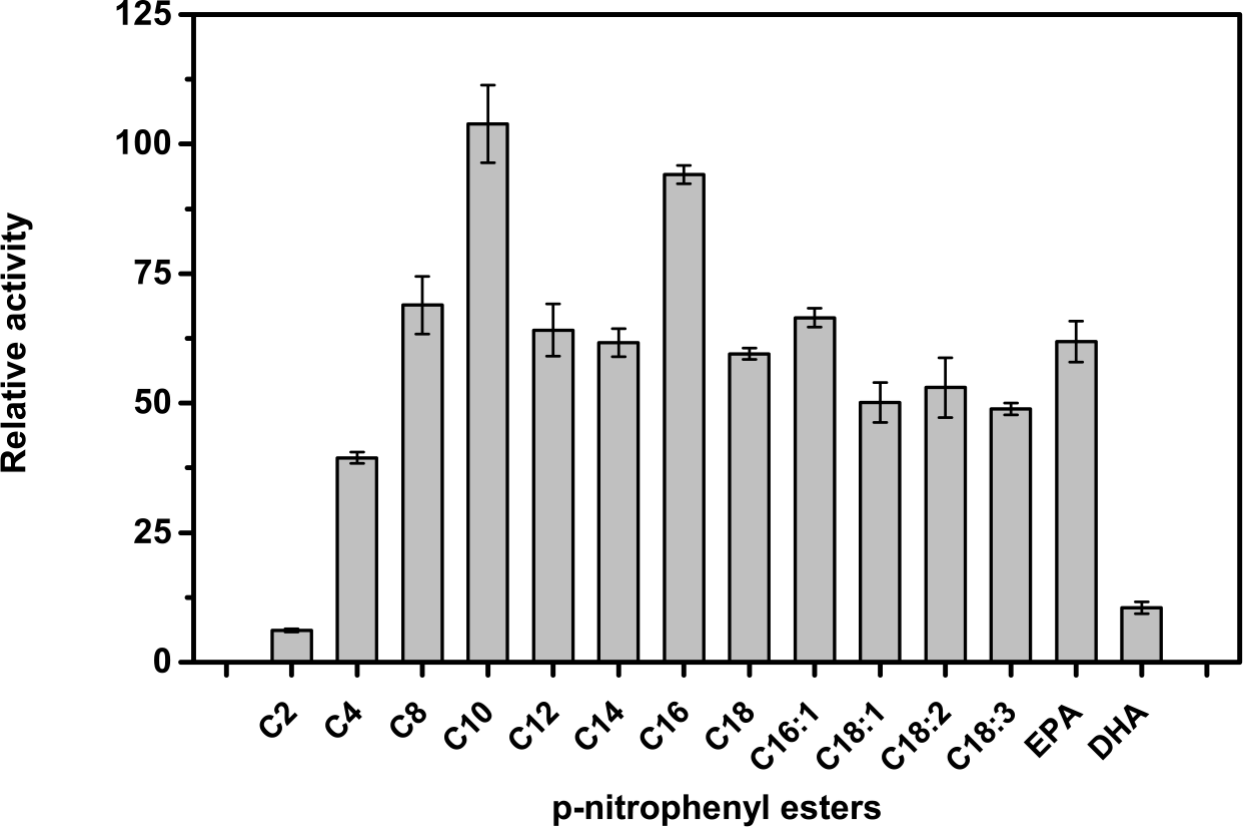
Phospholipase A1 activity towards *p*NP esters with different acyl chains. Activity of PLA1 on pNP-decanoate was taken as 100%.

PLA1 used in this study was isolated from *Thermomyces lanuginosus*(Lecitase^TM^) and is a recombinant enzyme preparation. This enzyme has been explored primarily for the degumming of vegetable oils [23,30]. However, its ability to hydrolyse triglycerides is only marginal in the vegetable oil degumming processes. Initially we have studied the esterase activity of PLA1 with p-nitrophenyl esters of fatty acids of various chain lengths. Some of the *p*NP esters were synthesised based on published methods [31,32]. Fig 1 shows that PLA1 was able to hydrolyse esters of chain length from C4–C20 with comparable efficiency. The activity was highest for C10 and lowest with C4 ester (40% of C10). The presence of unsaturation, single or multiple double bonds, did not significantly impact the esterase activity appreciably. However, esters of DHA were not efficiently hydrolysed. A similar chain length study was performed with lipase from *Bacillus subtilis* (BSL) and it was found that C8 chains were most efficiently hydrolysed [33]

### Hydrolysis of Anchovy oil by PLA1

**Fig 2:**
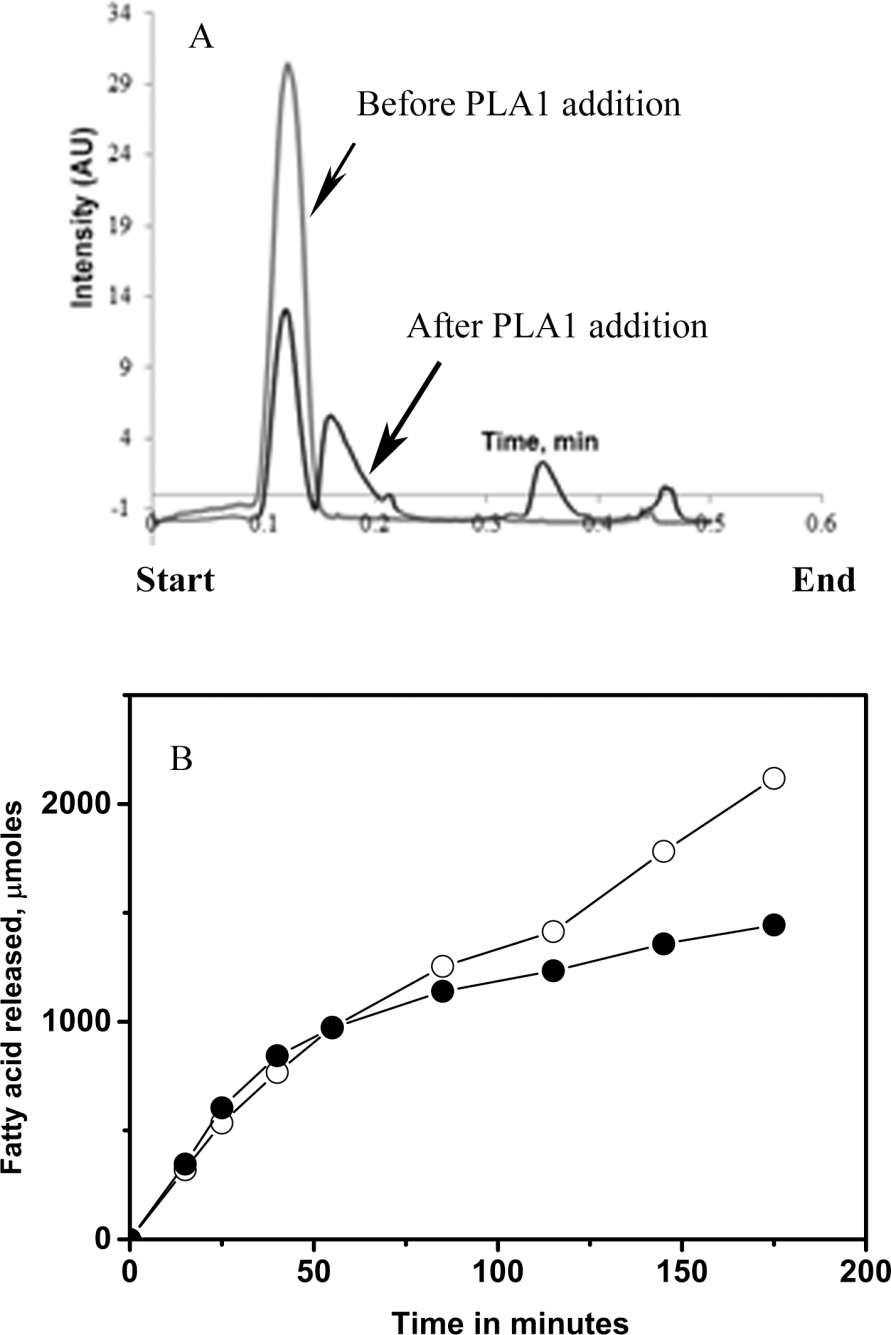
Time course of hydrolysis of anchovy fish oil by PLA1 and BSL. A, Iatroscan of unhydrolysed (light trace) and hydrolysed (darker trace) fraction of anchovy oil by PLA1. Start and the end of the scan are identified. B, pH stat based determination of rate of hydrolysis of anchovy oil in the presence of PLA1 (closed circle) and BSL (open circle).

**Fig 3:**
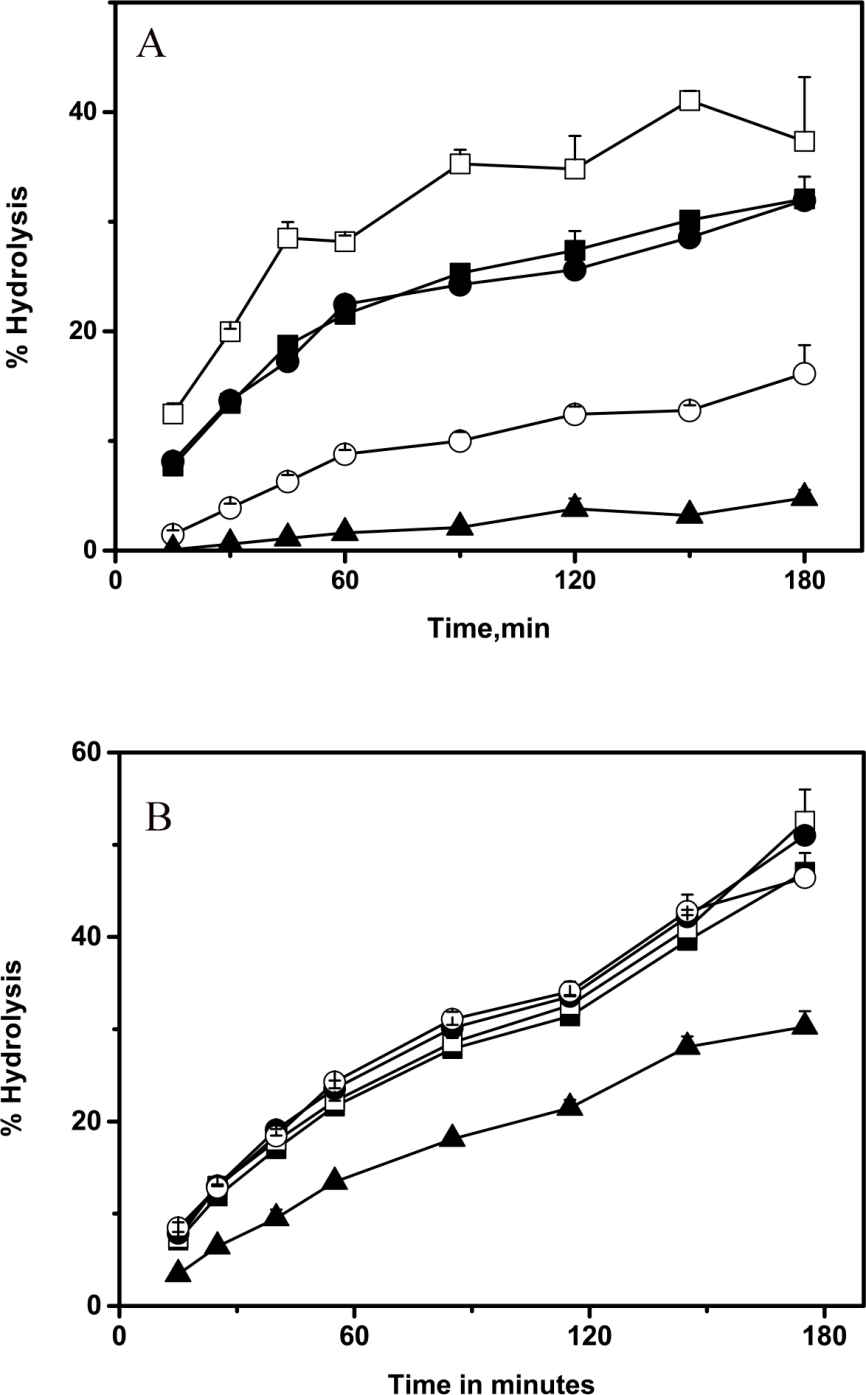
Time course of hydrolysis of anchovy oil and fatty acid distribution in the hydrolysate. Anchovy oil was subjected to hydrolysis by PLA1 (A) and BSL (B). The reaction product at various times was subjected to methylation and GC analysis. Percent hydrolysis of each fatty acid was calculated based on its hydrolysis at t_x_ as a fraction of t_o_. Saturated (□), Monounsaturated (●), EPA (○), DHA (▲), Total hydrolysis (■).

**Fig 4:**
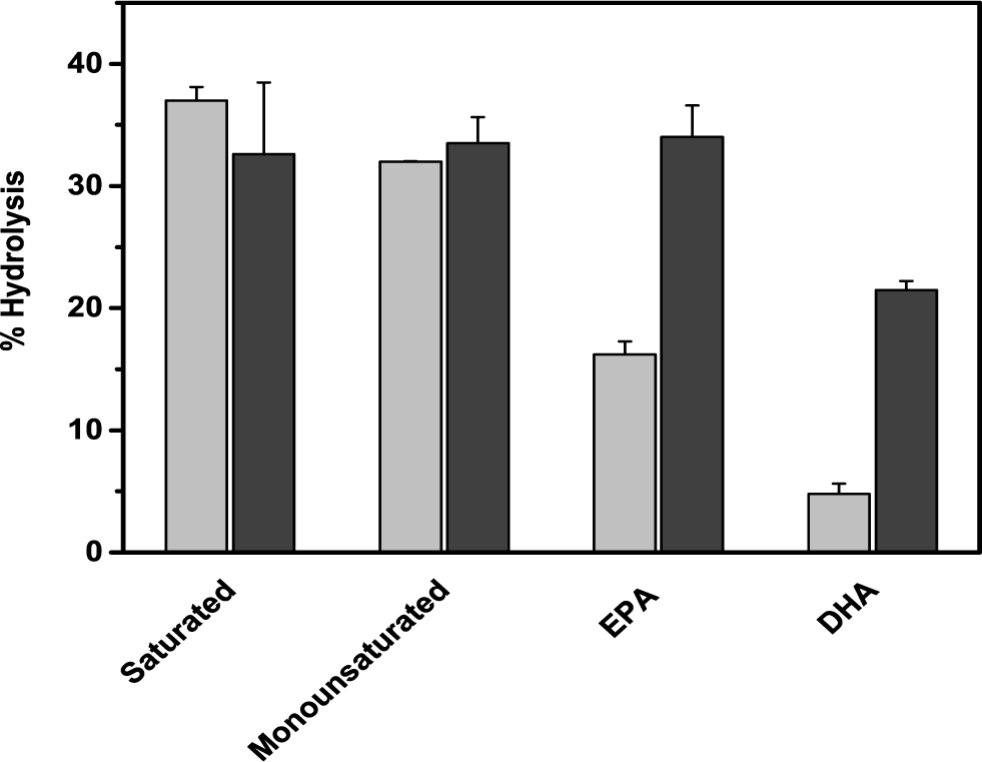
Percent hydrolysis of various fatty acids (from Fig 3) at 32% hydrolysis of anchovy oil by PLA1 (light) and BSL (dark). The relative proportions of fatty acids at other time points were also similar.

Initially hydrolysis of anchovy oil was tested in an oil–in-water emulsion and the extent of hydrolysis was monitored by Iatroscan. Fig 2A shows the scan before and after hydrolysis of the oil by PLA1. To obtain more quantitative kinetics of the hydrolysis, anchovy oil hydrolysis was monitored by a pH Stat, while maintaining a constant pH and with gum arabic (5%) as emulsifier. Initially we measured the rate of hydrolysis of anchovy oil by PLA1 and BSL (Fig 2B). Both enzymes were able to hydrolyse the oil at a comparable rate. At 37°C the extent of hydrolysis reached 45% by 2 h and then plateaued. The fatty acid fraction was separated from the glyceride fraction, methylated and analysed by GC. Fig 3A shows the time course of release of various fatty acids from anchovy oil by PLA1. Both saturated and monounsaturated fatty acids were extensively hydrolysed (approximately 40%of the total), while the percent hydrolysis of both the ω-3 fatty acids EPA and DHA was relatively poor 15% and 4%, respectively. Identical experiments were also performed with BSL (Fig 3B). BSL also hydrolysed oils with similar efficiency. However, the extent of hydrolysis of various fatty acids BSL was equal. While the extent of hydrolysis of saturated, monounsaturated and EPA were equal (50%), only 30% hydrolysis of DHA was observed. The fatty acid products obtained after 3h hydrolysis are shown in Fig 4. The data suggests that PLA1 discriminates against EPA and DHA, while BSL shows marginal discrimination against DHA. A significant five-fold higher discrimination of DHA by PLA1 compared to BSL was observed.

### ^13^C NMR studies of the anchovy oil hydrolysis by PLA1 and BSL

^13^CNMR spectra of complex triglycerides, such as fish oils, can provide positional information on the various fatty acids in the oil. This information enables a study of the positional specificity of fatty acid hydrolysis by PLA1 and BSL and also to obtain quantitative information on the extent of hydrolysis of fatty acids at each position. Initially we acquired the positional information of various fatty acids in anchovy oil. Table 1 provides the details of positional distribution of fatty acids in anchovy oil. The ratio of abundance of various fatty acids at sn-1,3 and sn-2 positions was observed to be 1.64 for saturated, 3.06 for monounsaturated, 4.2 for EPA and 0.6 for DHA. A ratio of 2 suggests the fatty acids are equally distributed. A ratio of 0.6 for DHA indicates it is predominantly present at the sn-2 position. The ratio is high (4.2) for EPA indicating its preferential distribution at sn-1,3 position, compared with the distribution of DHA.

**Table 1:** Positional distribution of various fatty acids in anchovy oil.

**Fatty acid**	**Distribution (%)**	
	**Sn-1,3**	**Sn-2**
Saturated	22.9	14.0
Mono unsaturated	18.7	6.1
Stearidonic acid (STA)	6.2	2.7
EPA	14.9	3.5
DHA	3.9	6.8

**Fig 5:**
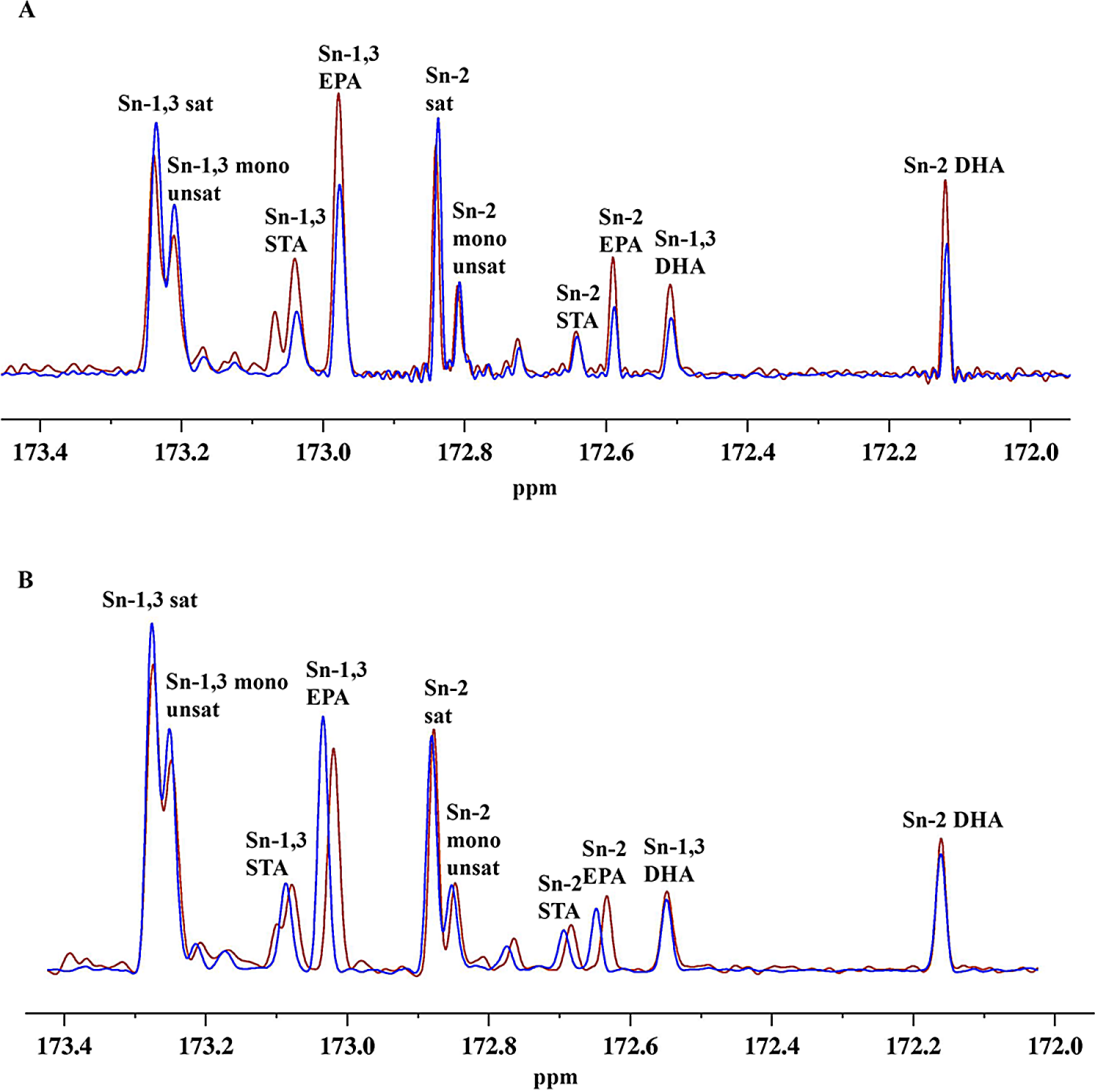
^13^C NMR spectra of oils: Spectra of the unhydrolysed oil fraction before (blue) and after (red) 30% hydrolysis of anchovy oil by PLA1 (A) and BSL (B). Fatty acids and their positions were marked. Sat (saturated), mono (monounsaturated), STA (stearidonic acid), EPA (eicosapentaenoic acid) and DHA (docosahexaenoic acid).

**Fig 6:**
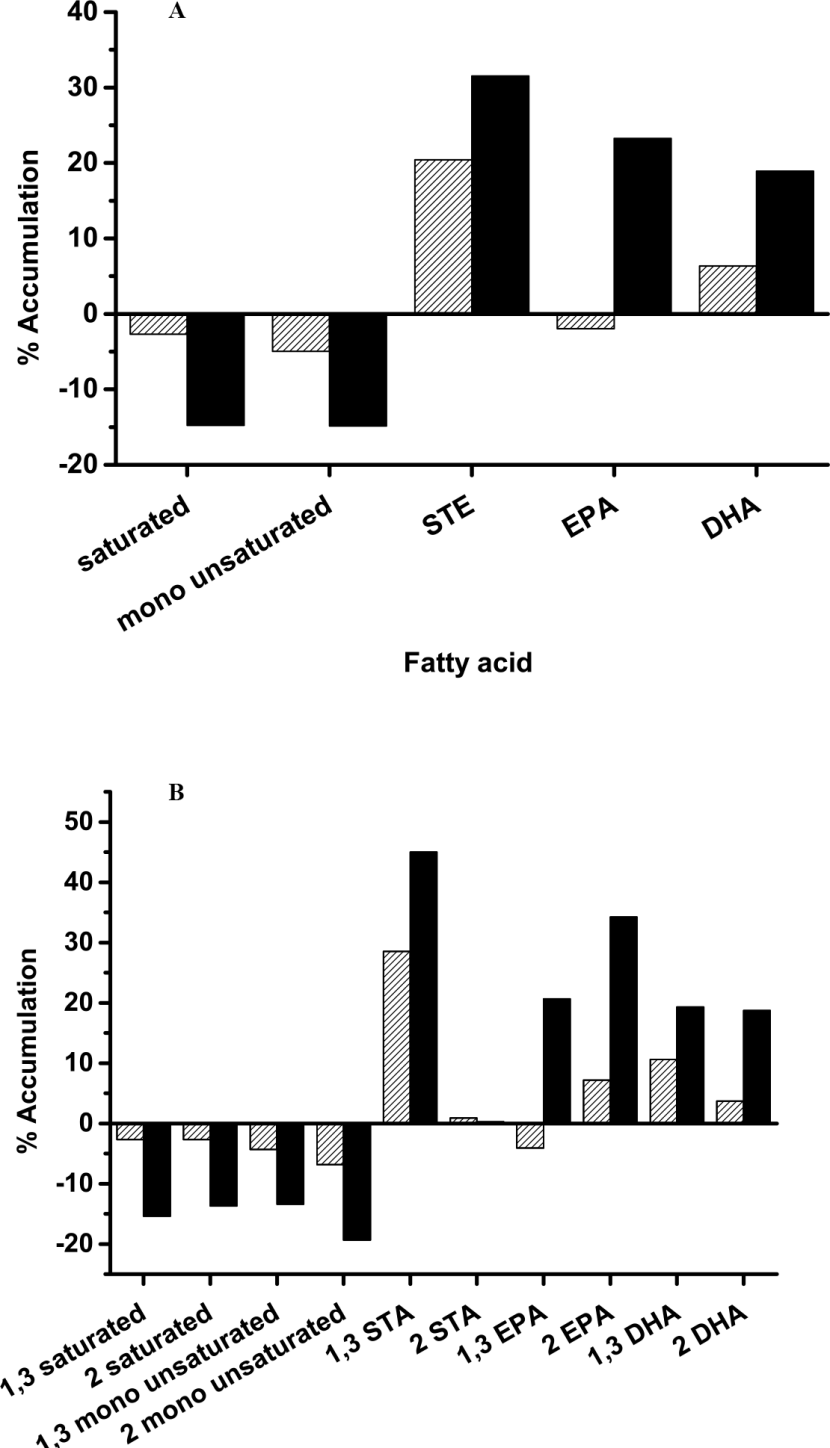
Percent accumulation of different fatty acid classes in triglyceride fraction after 30% hydrolysis of anchovy oil by PLA1 (dark) and BSL (light). A, Percent accumulation of each fatty acid is calculated as (^UH^FA_x_-^Total^FA_x_)/^Total^FA_x_) X 100). Each of the fatty acid fractions were calculated based on peak area in the NMR spectra. B, Percent accumulation data plotted with positional information.

**Fig 7:**
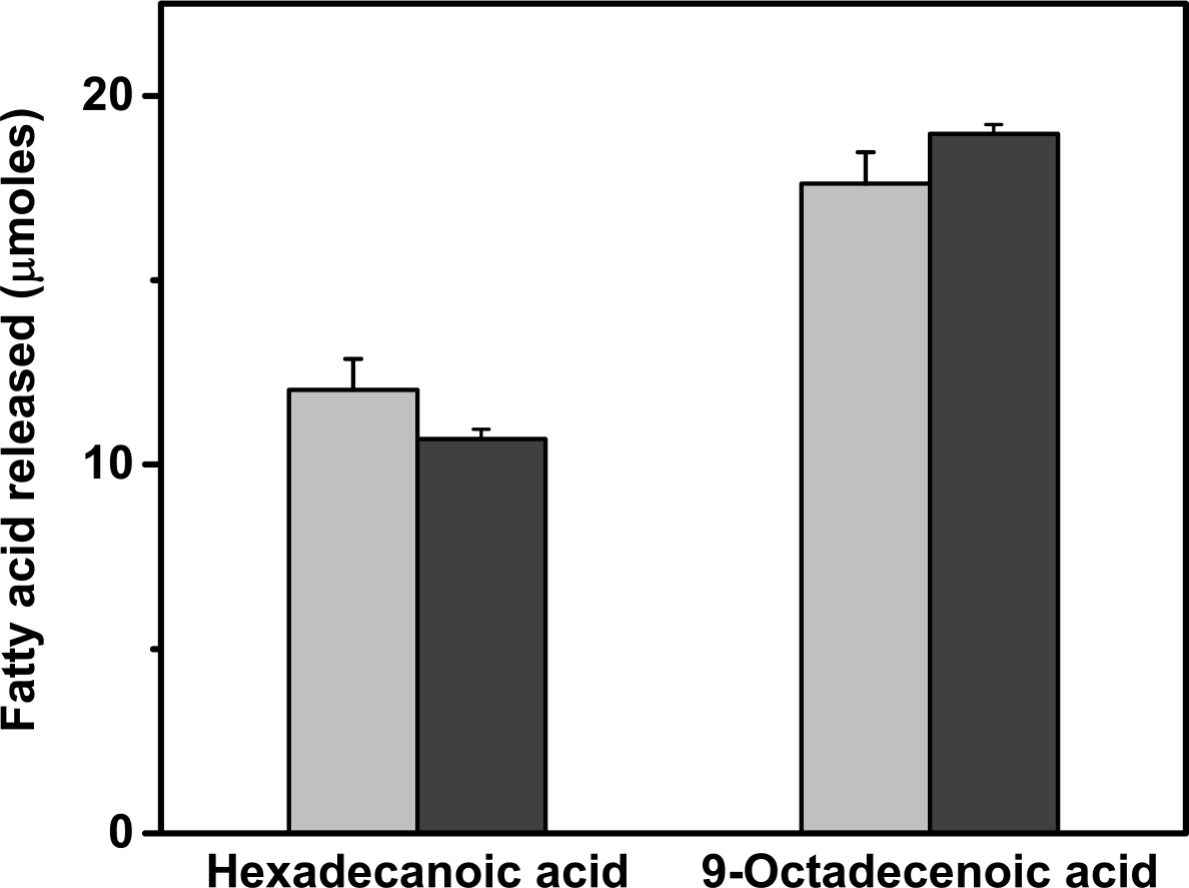
Lipase hydrolysis of structured triglyceride: 1,3-dioeoyl-2-myristic triglyceride was subjected to hydrolysis by PLA1 (Light) and BSL (Dark) and the released fatty acids were quantified by GC analysis.

Anchovy oil hydrolysis by PLA1 and BSL was allowed to proceed until 30% of the oil was hydrolysed and then the unhydrolysed portion of the oil was extracted and ^13^C NMR spectra were obtained. This process was repeated with unhydrolysed anchovy oil. Fig 5 shows the ^13^C NMR spectra of hydrolysed and unhydrolysed anchovy oil subjected to PLA1 (Fig 5A) and BSL (Fig 5B) treatment. The overlay of hydrolysed and unhydrolysed spectra for both PLA1 and BSL clearly demonstrates that PLA1 preferentially retains EPA and DHA more than BSL. Fatty acids remaining in the unhydrolysed portion of the oils, indicated as percent accumulation with PLA1 and BSL are shown in Fig 6. Saturated and monounsaturated fatty acids were preferentially hydrolysed by both the enzymes while ω-3 fatty acids were discriminated against. BSL also preferentially hydrolysed EPA over DHA. The percent accumulation data shown in Fig 6A indicates that EPA at sn-1 was more efficiently hydrolysed by BSL than by PLA1. BSL hydrolysed EPA at sn-1 but not DHA at sn-1or at sn-2, while PLA1 had no preference for EPA over DHA. These results indicate that the discrimination against ω-3 fatty acids by PLA1 is a result of the chemical nature of the fatty acid and not its position on the triglyceride. To verify this observation, we have performed hydrolysis of a structured triglyceride, 1,3-dioleoyl-2-hexadecanoic glycerol, using PLA1 and BSL and estimated the fatty acid products by GC (Fig 7). If PLA1 shows absolute preference for fatty acids at the sn-1,3 position, the hydrolysis of hexadecanoic acid at sn-2 should be much lower than that of oleic acid. PLA1 and BSL have hydrolysed each of the fatty acids almost equally, indicating that PLA1 did not show any specificity towards the sn-1,3 positions. However, there is a distinct preference against DHA in anchovy oil, indicating the fatty acid specificity of PLA1.

## Discussion

Enrichment of natural fish oils with ω-3 fatty acids is important for producing ω-3 concentrates with assured health benefits. Improving ω-3 fatty acid content in fish oils is challenging since the ω-3 fatty acids are asymmetrically distributed on two positions of glycerides (sn-1 and sn-3 are considered equal). Many lipases tested for improving ω-3 fatty acid content in glycerides were only partially successful since lipases during hydrolysis do not show strong specificity towards either the chemical nature of the fatty acids or to their positions on glycerides. We explored the utility of PLA1, a phospholipase with lipase activity, in enriching ω-3 fatty acids. Our study shows, based on ^13^C NMR position analysis, that PLA1 discriminates ω-3 fatty acids compared to the other fatty acids during hydrolysis.

### Phospholipases as TG hydrolases

PLA1 specifically hydrolyses *sn*-1 acyl esters from phospholipids and releases free fatty acids and lysophospholipids. PLA1 enzymes normally exhibit very little lysophospholipase and some lipase activity. PLA1 enzymes are descendants of neutral lipases, and several PLA1 sequences show substantial sequence similarity to the well characterized pancreatic, hepatic and endothelial lipases [18,34]. Although PLA1 enzymes are found in a wide variety of cells and tissues, only a small number of PLA1 enzymes were cloned and their substrate preferences are not well documented [18].

Lipase activity of phospholipases and *vice versa* was studied in few cases. The ratio of lipase to phospholipase activity of lipases or phospholipases varies widely and the ratio is not only related to the structure of the lipase/phospholipase but also depends on the reaction system [23].For example, PLA1 from *Thermomyces lanuginosus* used in this study shows hydrolytic activity against both triacylglcyerides and phospholipids and is used to degum oils [22,23]. PLA1, though has lipase activity, shows predominantly phospholipase activity at reaction temperatures above 40°C [23]. Pancreatic phospholipases hydrolyse phospholipids in the aqueous phase, while lipase from *Fusarium oxysporum* hydrolyses phospholipids in the oil phase [23]. Comparatively more structural studies were performed on lipases that also show phospholipase activity. Structural efforts were focused on the architecture of the active site and substrate preferences in lipases. The hydrophobicity and hydrophilicity balance of the β5, β9 loop and the lid domain of lipases play a selective role in preferring a triglyceride or a phospholipid [20,34,35]. The lid domain of guineapig pancreatic lipase related protein 2(GPLRP2) is reduced in size and consequent exposure of hydrophilic residues enables the active site to accept phospholipids and larger galactolipids [20]. Human pancreatic lipase that does not show phospholipase activity was not able to accommodate the phospholipid molecule. Sequence alignment of porcine pancreatic lipases indicates that Val260 is critical for interaction with lipids [36]. These studies suggest that volumes of the active sites in these enzymes play a critical role in substrate, i.e., glyceride or phospholipid, selection.

PLA1 from *Thermomyces lanuginosus*, used in this study, discriminated against DHA and partly against EPA when hydrolysing anchovy oil. In similar experiments using a lipase from *Bacillus subtilis* similar selectivity against EPA and DHA was not observed. Anchovy oil has DHA preferentially located at the sn-2 position (60%), compared to the sn-1,3 positions (40%). If PLA1 preferentially hydrolysed at the sn-1,3 position in oils then more DHA than EPA should be present in the unhydrolysed fraction. This was observed in our experiments (Fig 4). From analysis of fatty acid preference during hydrolysis by PLA1 it is apparent that PLA1 poorly hydrolysed DHA. However, we did not observe such positional or chemical specificity in BSL mediated hydrolysis. Conformationally ω-3 fatty acids show limited states due to the presence of multiple double bonds compared to saturated or monounsaturated fatty acids. This aspect may strongly influence their binding to the active sites of enzymes.

The position specific hydrolysis of anchovy oil by PLA1 and BSL (Fig 6B) suggests that ω -3 fatty acids were discriminated against and saturated and monounsaturated fatty acids were preferentially hydrolysed. The position at which these fatty acids were present did not influence their hydrolysis. To confirm the positional specificity of these hydrolases we have employed a structured glyceride with fixed positional distribution of fatty acids. Using1,3-dioleoyl-2-hexadecanoic glycerol, a structured TG, neither PLA1 nor BSL exhibited any position specific hydrolysis under our reaction conditions, with both fatty acids being equally hydrolysed. In a previous study, five lipases were tested for their specificity by using fish oil and methyl esters of EPA, DHA and palmitic acid. All lipases discriminated against EPA and DHA when presented as methyl esters. However, lipase from *Thermomyces sp*. has shown discrimination against DHA, particularly in the early stage of the hydrolysis reaction, while lipase from *Candida rugosa* was most efficient in the enrichment of DHA in the glyceride fraction [6]. In another study lipase from *Candida rugosa* discriminated ω-3 fatty acids from other fatty acids during hydrolysis of sardine oil [37]. These studies also suggest the specificity in hydrolysis of natural oils by lipases or phospholipases is dependent on the temperature and duration of reaction. Absence of positional information on fatty acids can confound interpretations on selectivity by these enzymes.

Our study and similar studies with lipase from *Thermomyces lanuginosus* suggests that hydrolysis is due to fatty acid selectivity more than regioselectivity [8]. Several reports on fatty acid specificity of lipases and phospholipases, listed above and including our study, and also structural information on these enzymes suggests that the active site requirements of SFA/MUFA vs. polyunsaturated fatty acids are significantly different. Observed linear channels near the active sites of lipases for binding of fatty acids are probably less suited to bind polyunsaturated fatty acids, such as ω-3 fatty acids. A related previous study showed that substrate binding was dependent on the relative volume of the substrate to the volume of the active site [38]. Thirty eight lipases were investigated *in silico*, for their affinity to structured TGs with different chemical and positional compositions. This study indicated that binding affinity differences between various substrates with lipases is complex, but helped to identify active site positions that are critical to binding. This information could be useful for designing site saturated mutagenesis to identify amino acid substitutions that would enhance hydrolysis of specific fatty acids. Few studies have successfully identified amino acid positions that are important in binding triglycerides and phospholipids [20]. Further studies are required to enable the design of enzymes that can enrich ω-3 fatty acids.

## Conclusion

The fatty acid position selective hydrolysis of PLA1 was tested for the hydrolysis of anchovy oil. Fatty acid chain length selectivity of PLA1 was investigated using *p*NP esters of chain length C2 to C22, including the long chain PUFAs, EPA and DHA. For anchovy oil hydrolysis SFAs and MUFAs were preferentially hydrolysed by PLA1 and ω-3 fatty acids were discriminated against. Of the ω-3 fatty acids, EPA was more preferentially hydrolysed than was DHA. Lipase from *Bacillus subtilis* did not show discrimination of fatty acids to the same extent as PLA1. Hydrolysis of the structured triglyceride, 1,3–dioleoyl-2-hexadecanoic glycerol, supported that the discrimination property of PLA1 was primarily due to the chemical nature of the fatty acids rather than its position in the triglyceride. Our study suggests that PLA1 is a potential catalyst for selective enrichment of ω-3 fatty acids in triacylglycerides.
